# Neurocognitive Precursors of Substance Misuse Corresponding to Risk, Resistance, and Resilience Pathways: Implications for Prevention Science

**DOI:** 10.3389/fpsyt.2019.00399

**Published:** 2019-06-14

**Authors:** Emma Jane Rose, Giorgia Picci, Diana H. Fishbein

**Affiliations:** ^1^Program for Translational Research on Adversity and Neurodevelopment (P-TRAN), The Edna Bennett Pierce Prevention Research Center, The Pennsylvania State University, University Park, PA, USA; ^2^Department of Human Development and Family Studies, The Pennsylvania State University, University Park, PA, USA

**Keywords:** neurocognitive, neuroimaging, substance misuse/abuse, risk, resilience, resistance, prevention science

## Abstract

Studies of substance misuse prevention generally focus on characteristics that typify risk, with the assumption that the prevalence of the problem will be optimally reduced by identifying, targeting, and reducing or eliminating risk factors. However, this risk-centered approach neglects variations in individual-level and environmental characteristics that portend differential pathways that are distinguishable by timing of substance use initiation (e.g., early versus delayed), the likelihood of use escalation versus eventual desistance, and enduring abstinence, despite exposure to significant risk factors. Considering the various underpinnings of these distinct substance use trajectories is critical to a more nuanced understanding of the effects, potency, and malleability of factors that are known to increase risk *or* confer protection. Here, we discuss three pathways relative to substance use patterns and predictors in the context of adversity, a well-known, highly significant influence on propensity for substance misuse. The first pathway is designated as “high risk” based on early onset of substance use, rapid escalation, and proneness to substance use disorders. Individuals who defy all odds and eventually exhibit adaptive developmental outcomes despite an initial maladaptive reaction to adversity, are referred to as “resilient.” However, another categorization that has not been adequately characterized is “resistant.” Resistant individuals include those who do not exhibit problematic substance use behaviors (e.g., early onset and escalation) and do not develop substance use disorders or other forms of psychopathology, despite significant exposure to factors that normally increase the propensity for such outcomes (e.g. trauma and/or adversity). In this paper, we apply this conceptualization of risk, resistance, and resilience for substance misuse to a more fine-grained analysis of substance use pathways and their corresponding patterns (e.g., non-use, initiation, escalation, desistance). The significance of the progression of neurocognitive functioning over the course of development is discussed as well as how this knowledge may be translated to make a science-based determination of intervention targets. This more encompassing theoretical model has direct implications for primary prevention and clinical approaches to disrupt risk pathways and to optimize long-term outcomes.

## Introduction

Adolescents who initiate substance use and later develop substance use disorders (SUDs) transition through multiple sequential stages, including experimental or social use, escalation of use, maintenance, abuse, and eventual dependence (1, 2). However, a linear progression along this pathway is not often realized, with individuals showing considerable variability in the likelihood of early, experimental use and significant fluctuations in patterns of usage, escalation, and desistance (3, 4). For example, there are subgroups of users who may never escalate, maintaining nondependent use for decades. While some exhibit intermittent periods of cessation or abstain permanently, others rapidly escalate and go on to develop SUDs. Discriminating between these different user types and delineating which individuals are more likely to follow different pathways is key to identifying critical windows of opportunity for preventing substance misuse.

A potent risk factor influencing the transition from social/experimental use to problematic use and eventual dependence is the experience of traumatic and other chronic or severely stressful events in childhood (5, 6). Indeed, exposure to adversities such as child maltreatment, poverty, and witnessing or experiencing violence have been repeatedly implicated in trajectories leading to SUDs (7–9). The literature is replete with studies documenting the impact of early adversity on neurocognitive development throughout childhood and adolescence and, in turn, how adversity-related deficits or delays in neurocognitive function in youth can increase vulnerability to a myriad of risk behaviors, such as substance misuse (10–12). Integrity of neurocognitive development translates to the ability to self-regulate behavior and emotion via “top-down” cognitive control over affective responses to life’s challenges. The development of these processes may be particularly influential in adaptations to adversity. Thus, variations in neurocognitive trajectories are likely more pronounced in populations where adversity prevails, which, in turn, may correspond to a wide range of behavioral pathways and outcomes, from low to high risk (13–15). In other words, adversity can result in diverse outcomes (*multifinality*) depending largely upon the ways in which the nervous system is affected in exposed individuals.

Substance use outcomes in response to adversity, including its impacts on the brain, may manifest in the following general developmental pathways: *risk* (initial and sustained reactions to adversity, resulting in maladaptive outcomes), *delayed risk* (apparent early resistance to adversity but eventual decline toward maladaptive outcomes), *resilience* (initial reaction followed by gradual degradation of response to adversity with eventual restoration of adaptive developmental outcomes), and *resistance* (absence of change in developmental trajectory despite exposure to adversity). Developmental periods that correspond with these patterns may include an initial departure in direction (e.g., risk vs. resistance), the time point at which trajectories may diverge (e.g., resistance vs. delayed risk *or* risk vs. resilience), and the time beyond which specific risk outcomes emerge (e.g., substance abuse). Developing more precision-based interventions will require a clearer delineation of critical time points when influential factors in substance misuse act on emergent neurocognitive systems in a manner that increases the likelihood of following one of these pathways versus another.

**Figure 1:**
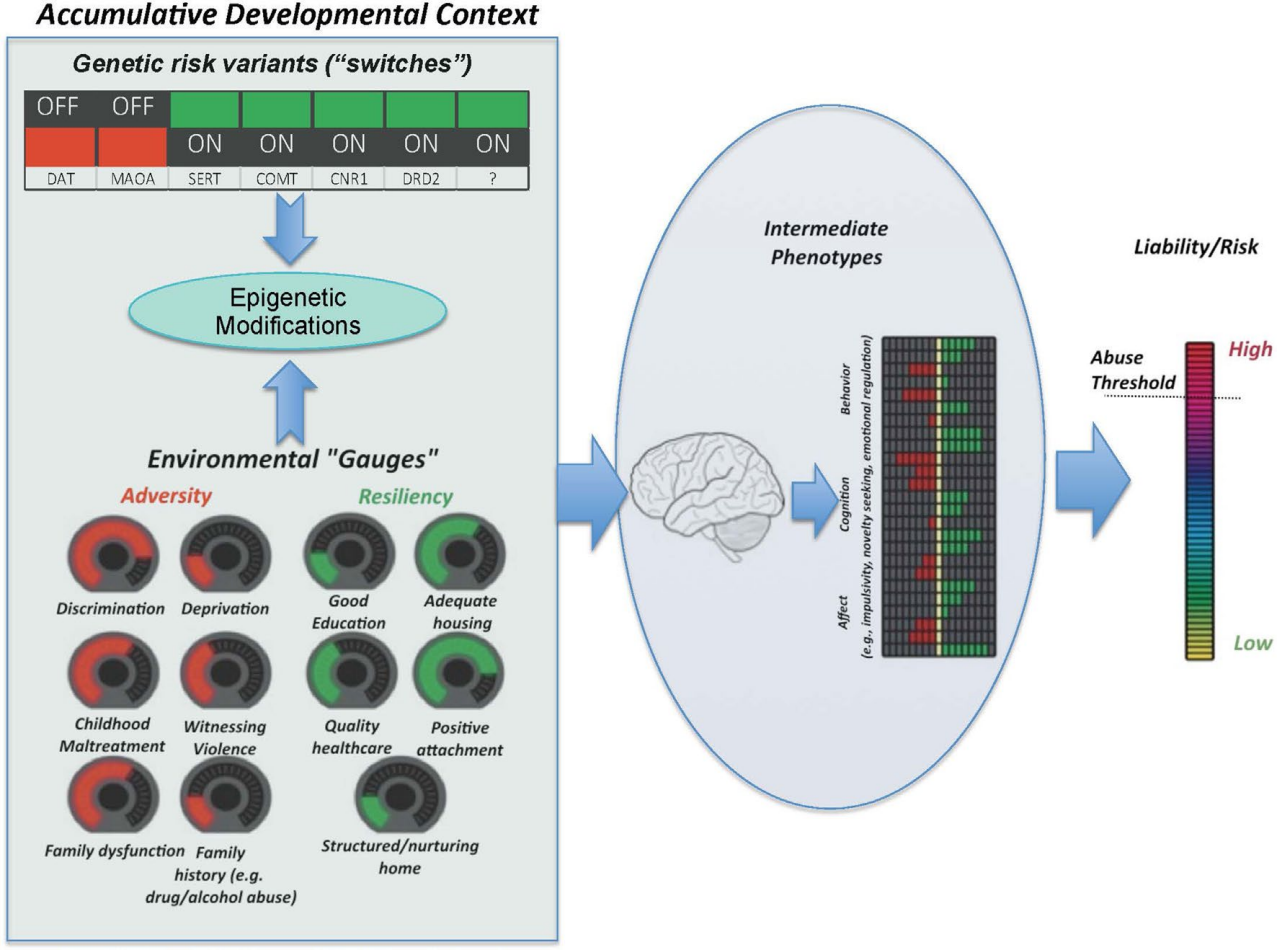
The Accumulative Risk Model. Shown here are the two main categories of factors that constitute the accumulative developmental context, i.e., genetic and environmental factors. The combined effect of the number, type, and severity of these factors confers risk for substance abuse. Genetic variants are considered as switches, which are either “on” or “off.” This conceptualization reflects the common binary consideration of genetic risk (i.e., individuals are often considered at risk or not depending on the particular variant of a given gene that they happen to carry). To reflect their more continuous nature, environmental factors are presented as dials, turned up or down depending on the magnitude of the experience. The unique combination of genetic switches and environmental dials drives neurodevelopmental trajectories that underlie particular cognitive, behavioral, and affective intermediate phenotypes, which, in turn, can result in an increased liability threshold, beyond which an individual is considered to be at greater likelihood of developing problematic substance use behaviors and eventual SUD. Importantly, the functional relationship between factors is not linear, and some environmental factors may exacerbate or attenuate the effects of the particular genes via epigenetic modifications.

As described herein, our *Accumulative Risk Model* (see **Figure 1**) depicts the interactive influence of genetic risk markers and environmental contexts (both detrimental and protective) on intermediate phenotypes, including distinct or interwoven cognitive, affective, and behavioral trajectories and associated neural factors (i.e., variability in brain structure and function) that underpin pathways for outcomes ranging from adaptive to maladaptive. In our model, the dynamic interplay of factors in the developmental context exerts differential impacts on these intermediate phenotypes and their neurobiological substrates in a manner that is contingent upon developmental stage. As such, missing time-dependent opportunities to intervene and redirect development translates to a higher probability of individuals exceeding a liability threshold for high risk behaviors, including substance misuse. In this paper, we review the evidence in support of this integrative framework and its relevance to the ability of evidence-based prevention programming to strengthen these neurodevelopmental processes, thereby attenuating negative effects of risk factors and reinforcing resilience and/or resistance. Such a science-based strategy has potential to redirect developmental pathways away from risky behaviors such as substance abuse.

The content presented in this review was selected via a nonsystematic/narrative review process, whereby we searched standard sources (e.g., PubMed; www.ncbi.nlm.nih.gov) for relevant but broad terms. This included various combinations of the following terms: substance abuse/misuse; SUD(s); development; risk; resilience; genetics; environment; and prevention/intervention. In addition to the articles that resulted from these searches, we engaged in an iterative process by which relevant publications that were cited in specific articles were also included in our review.

### The Accumulative Risk Model

#### Defining Risk: The Accumulative Developmental Context

Risk is commonly thought of as binary and deterministic, as reflected in the tendency to designate individuals as either “at risk” or not, and the assumption that those who are “at risk” are more likely to assume a maladaptive pathway, characterized by high-risk behaviors such as substance abuse. However, risk is better conceptualized as a continuous trait—*liability*—with values ranging from low to high. An individual’s positioning along the liability continuum is determined by a number of pertinent intermediate phenotypes—such as patterns of behavior, cognition, and affect—modulated by an individual’s unique brain structure and function. This neurobiological variability is, itself, a function of a highly complex and individualized range of factors, including those that are potentially malleable, such as environmental and contextual conditions, and those that are not so amenable to change or manipulation, such as genetic factors.

The constellation of factors that confer adaptive or maladaptive neurodevelopmental trajectories can be conceptualized as the “accumulative developmental context.” Within this context are factors that either increase (i.e., risk factors) or decrease (i.e., protective factors) liability (**Figure 1**). Importantly, the number and type of risk and protective factors are presumed to be unique between individuals and the interplay between factors determines an individual’s level of liability more so than any one factor alone. The influence of each factor, whether risk or protective, is not necessarily linear and some factors may act as moderators of other relevant factors, either amplifying or decreasing their risk or protective potential. Understanding these relationships and how the accumulative developmental context increases liability for SUD or, alternatively, offers protection and fosters resilience or resistance, promises to provide critical information on which to base the development of approaches to prevent SUDs.

### Putatively Distinct Developmental Trajectories

**Figure 2:**
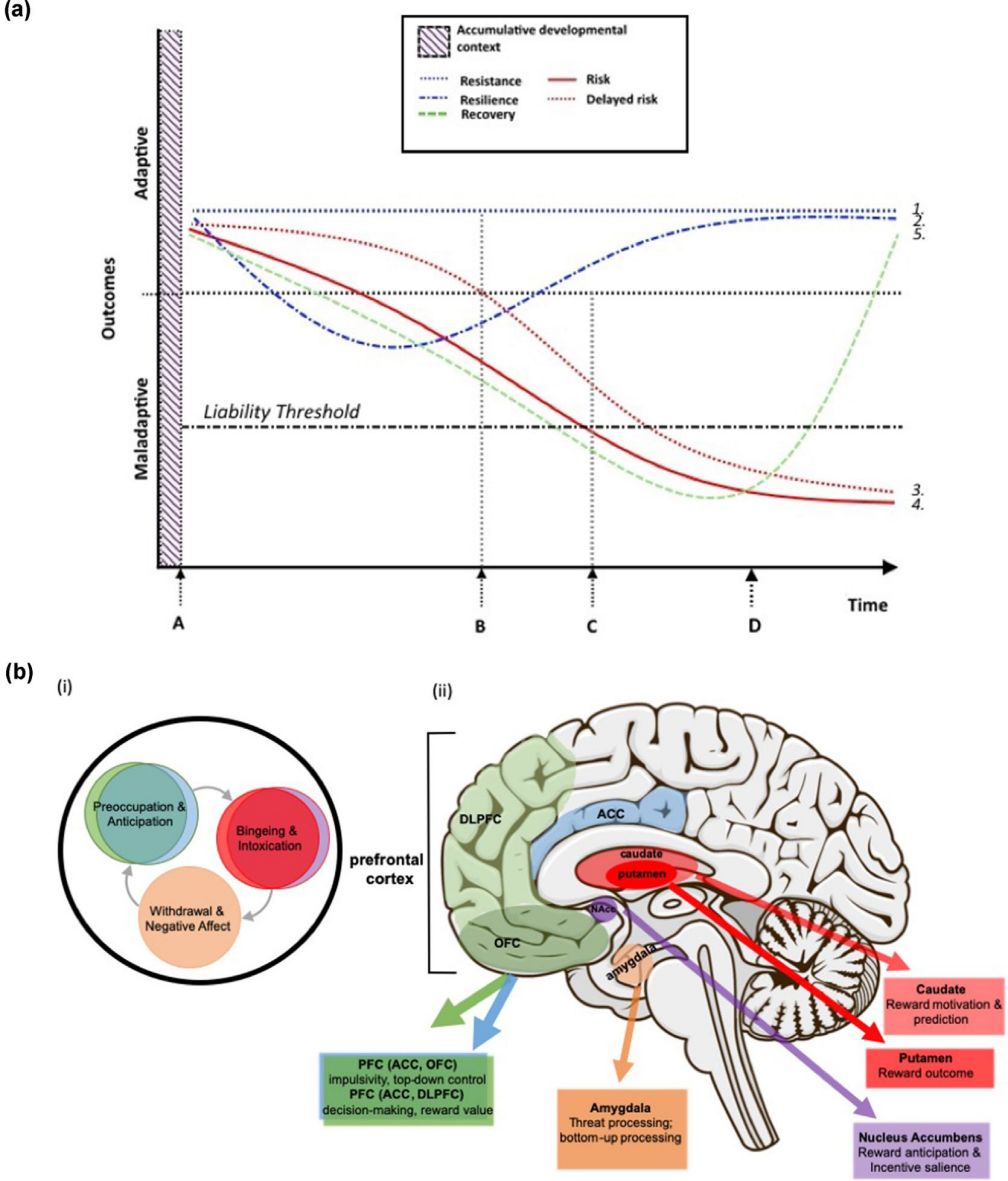
**(a)** Theoretical neurodevelopmental trajectories corresponding to adaptive and maladaptive outcomes. **(1)** *Resistance*: absence of change in the developmental trajectory despite exposure to adversity; **(2)** *Resilience*: initial reaction followed by gradual degradation of the response to adversity and eventual restoration of an adaptive developmental trajectory; **(3)** *Delayed risk*: apparent early resistance to adversity but eventual decline toward maladaptive outcomes/risk; **(4)** *Risk*: initial and continued reaction to adversity, resulting in maladaptive outcomes, and **(5)** *Recovery*: a shift in neurodevelopmental trajectory back toward adaptive outcomes, following disease onset/crossing the liability threshold for a disorder, and corresponding to intervention (i.e., treatment) onset. Critical time points in delineating those factors that contribute to risk or resistance and resilience include **(A)** the initial departure in neurodevelopmental trajectories, perhaps corresponding to adversity or other stressors, **(B)** the time point at which trajectories may deviate from initial direction (i.e. resilience and delayed risk), **(C)** the time beyond which specific risk outcomes (e.g., substance abuse) are highly probable and beyond which individuals with high levels of risk are likely to have crossed the liability threshold, and **(D)** intervention/treatment onset. Note: “outcomes” includes all relevant intermediate phenotypes consisting of or related to neurodevelopment (e.g., brain structure and function, cognition, behavior, affect, etc.). The “accumulative developmental context” refers to the combined genetic and environmental context that drives brain development (as depicted in **Figure 1**), and although this context is critical to neurodevelopment it precedes observable distinctions between neurodevelopmental trajectories; this includes those factors that may be considered to be detrimental or protective. **(b)** **(i)** Key stages in the cycle of addiction (after 22) and **(ii)** brain regions that these key stages map to and putative functions of each region that are relevant to the development of SUD. Included here are critical regions in which functional and structural deficits have been shown to be associated with at least one of the stages in the cycle of addiction. Functional variability in these regions in response to the characteristics of the accumulative developmental context (i.e., key genetic, environmental, and psychosocial influences on neurodevelopment) likely underlie the likelihood of which trajectory (i.e., from 2a.) an individual follows. If so, these same regions and the cognitive, behavioral, and affective functions they support have considerable potential to serve as targets for preventive interventions.

Liability for high-risk behaviors or other suboptimal outcomes is commonly considered from the perspective of being either at risk or resilient, with the corresponding assumption being that either trajectory is strongly associated with the prevalence (or sheer number) of risk or protective influences, respectively (16–19). While there is a wealth of experimental evidence to support this characterization, possible developmental pathways arising from any given context includes a range of potential positive and negative trajectories (20, 21) (see **Figure 2a**). Pathways that lead to maladaptive outcomes include the typically considered “risk” pathway; i.e., adverse external conditions and the development of suboptimal intermediate phenotypes, together that increase an individual’s likelihood of crossing the liability threshold for high-risk outcomes, such as SUD and other psychopathologies. Typically, risk is described as occurring in close temporal proximity to the factors that promote its expression (e.g., changes in cognitive or behavioral functioning that more or less immediately follow some stressful life event). A related, but not commonly considered, negative trajectory is “delayed risk,” which occurs when there is a temporal delay or disconnect between the factors that promote a high-risk trajectory and the observable changes the portend a maladaptive outcome. Though not often distinguished in the literature, determining those aspects of the developmental context that confer risk vs. delayed risk may be helpful in the design of preventive interventions. In particular, such information may lead to programs aimed at individuals who may not immediately appear to be at risk but for whom early evidence-based intervention may be particularly advantageous (i.e., potentially stemming the proliferation of maladaptive phenotypes).

At the positive end of the spectrum are resilience and the related, but theoretically distinct, concept of “resistance.” Resilience can be defined as the later expression of adaptive/optimal outcomes despite initially exhibiting negative responses to challenging or threatening circumstances (e.g., adversities and traumas, such as poverty, maltreatment, violence). Resilience-related factors are those that enable an individual to rebound from adversity- or trauma-related dysfunctions or deficits and to achieve their original state or otherwise adaptive outcome(s). In contrast, *resistance* is characterized by the maintenance of the original state despite exposure to stressful events or contexts; i.e., developmental pathways remain unaltered despite significant stress/trauma. A third possible positive trajectory—“recovery”—involves the resumption of function following the development of a maladaptive outcome, such as SUD, and subsequent intervention/treatment (**Figure 2a**). Although possibly driven by the same or similar factors as resistance and resilience (e.g., more optimal levels of neurocognitive functioning or emotional regulation), it is probable that recovery is at least partially distinct in terms of the pathway itself, the factors that promote it, and the timing (i.e., only following intervention). As such, recovery may constitute a third distinct class of positive adaptation. In support of this notion, and in the context of SUD specifically, recovery is highly likely to be distinguishable from resistance and resilience since SUD-related neuroadaptations may not be reversible (23); thus, individuals who recover from SUD do so without regaining a substantial degree of original functioning. Instead, other compensatory mechanisms may facilitate overall functioning in a way that is adaptive and allows individuals with SUD to achieve recovery and avoid relapse (24–26).

## Putative Underpinnings of Distinct Developmental Trajectories

Determining which experimental or social substance users will progress to abuse (i.e., a maladaptive pattern of substance use manifested by recurrent and significant adverse consequences) and dependence (i.e., a chronic relapsing brain disease characterized by compulsive drug seeking despite harmful consequences) is a longstanding question that has compelled researchers and practitioners to better understand, predict, and effectively intervene in maladaptive patterns of substance use. As depicted in the Accumulative Risk Model, the interplay between an individual’s genetics and their environmental and contextual experiences during critical periods of development give rise to patterns of neurobiological functioning, stress physiology, personality/temperament, and emerging coping strategies that determine the individual’s response to the prevailing social and environmental conditions. The nature of this response contributes to eventual substance use outcomes, including whether an individual will or will not engage in substance use and whether use will progress to abuse and dependence. A critical step in delineating the distinct etiological pathways under consideration here is understanding how relevant person-level characteristics predict or moderate outcomes and interact with environmental influences in unique and complex ways to either promote or preclude substance misuse.

### Neurocognitive Pathways to Substance Misuse

As noted above, there are commonalities in the key factors (risk and protective) that give rise to particular types of substance use pathways (i.e., adaptive or maladaptive); distinguishing between those that are more tightly coupled to one specific pathway (i.e., risk, delayed risk, resilience, or resistance) is not possible based on current knowledge and given limitations of the extant research. For example, most studies consider outcomes as either positive/adaptive or negative/maladaptive (e.g., having an SUD or not) at a single time point and lack the longitudinal perspective and temporal specificity needed to distinguish the putative pathways under consideration here. Nonetheless, defining the differential constellations of influences that lead to distinctive pathways toward or away from substance abuse is a paramount task; one holds considerable potential to lead to more personalized interventions with potential for population level impacts. Working backwards within the Accumulative Risk Model, from cognitive and behavioral phenotypes to their more basic substrates, risk and protective factors that cross trajectories are described briefly below. The following subsections consider evidence that implicates neurocognitive factors in the four divergent pathways under consideration here (i.e., risk, delayed risk, resistance, and resilience).

#### Risk for Substance Misuse

##### Genetic Vulnerabilities

There have been many genetic risk studies for SUDs that have delineated gene variants that appear to be associated with specific types of abuse (e.g., alcohol/alcohol dehydrogenase genes; nicotine/cholinergic receptor genes; opiates/opioid receptor mu genes). However, individual gene variants do not necessarily increase risk of using or abusing *specific* substances and, indeed, there is evidence that certain genes impact neurobiological systems and phenotypic traits in a manner that may directly influence pathways toward or away from substance use more *generally* (27). This includes genes that are involved in stimulus–reward processing pathways in dopaminergic (e.g., *DRD2, MAOA, COMT*), serotonergic (e.g., *HTR3A, HTR1B, HTR3B*), GABAergic (e.g., *GABRA1, GABRA2, GAD1, KCNJ9/GIRK3*), and glutamatergic neurotransmission systems [e.g., *GRIN2C*; see Ref. (28) for a review]. The phenotypic traits that are associated with these types of “risk” genotypes (e.g., high reward sensitivity, high impulsivity, low risk aversion, a tendency toward compulsive drug seeking) fundamentally interact with stress exposures that, when repeated and/or severe, have potential to compromise the development of neural systems that underlie social, behavioral, cognitive, and emotional functioning in profound and enduring ways (29, 30).

Genetic vulnerabilities in combination with the developmental stage(s) of exposure are critical to the differential effects that the exposure to stress can have on social, psychological, and neural functioning and, in turn, risk for substance abuse (31, 32). Genetic variations contribute an individual’s response to existing social influences; thus, genetic influences on propensity to substance abuse and dependence are thought to mediate or moderate the impact of environmental factors on individual characteristics that are associated with risk, with stress exposures being particularly impactful (33). At the core of the gene-by-environment interaction are epigenetic modifications that occur at the level of gene functionality in response to changes in the environment. Adverse experiences, especially in early life, have potential to modify gene expression or suppression with important implications for phenotypic impact on stress hormones and behavior (34, 35). Ongoing environmental change can further modify epigenetic processes, for better or for worse, helping to explain individual differences in response to stress as well as the potential for positive environmental change (e.g., intervention) to reverse earlier negative modifications. Thus, as indicated in our conceptual model (**Figure 1**), not all who are exposed to stress and/or trauma will exhibit maladaptive physiological and psychological stress responses that affect substance abuse liability; differential susceptibility to this outcome is a function of the complex interrelationships among genetic, environmental, and epigenetic factors that individuals dynamically experience.

##### Environmental Influences

As noted in **Figure 1**, there are a variety of environmental factors that can influence developmental trajectories in a manner that increases the risk for substance misuse. Of particular relevance here are those factors that we know promote adaptations of relevant neurodevelopmental pathways such that an individual’s liability for substance abuse and/or dependence are substantially increased. Contextual factors known to interact with biological factors to increase SUD liability include social and cultural systems, stress, and trauma (36).

Childhood maltreatment (CM) is a particularly potent risk factor for substance abuse and dependence (6). Those who experience CM initiate illicit substance use twice as often as nonmaltreated peers and are more likely to abuse substances earlier in adolescence (5, 37). Moreover, an estimated 40–50% of individuals who experience this type of trauma will develop a substance abuse problem in their lifetime (5). Neurobiological changes at the level of brain structure and function have been shown to underlie both CM and SUD and are often found in overlapping brain regions and networks (38–42). Disentangling the specific contributions of CM *per se*, versus those changes that arise in response to early and sustained abuse of substances, presents an interesting and important challenge. Although more research is needed in this domain to understand the independent, interactive, and potentially synergistic, contributions of CM and SUD to neurodevelopmental trajectories in young people, a recent review of the neurocognitive evidence of neurobiological pathways underlying SUD risk provides support for CM-related alterations in three interconnected systems that may heighten SUD vulnerability (**Figure 2b**), (1) *reward processing*—ventral striatum, anterior cingulate cortex (ACC), mPFC (including OFC) and amygdala; (2) *executive cognitive function (ECF)*—prefrontal cortex (PFC), including dlPFC and mPFC; and (3) *threat processing*—medial temporal lobe, in particular the amygdala (43).

Poverty is another common and particularly potent environmental influence to consider when delineating the neural pathways underlying SUD risk. There is consistent evidence to suggest that a child’s socioeconomic status (SES) is predictive of neurocognitive trajectories across development and longer-term outcomes, such as academic achievement (44), with lower SES children experiencing suboptimal or maladaptive developmental trajectories, including neurodevelopmental pathways (32, 45–48). The most consistent *structural* impacts of poverty/low SES are seen in brain areas and processes that are sensitive to the effects of stress, including those that are relevant for SUD risk (e.g., hippocampus/memory; amygdala and medial temporal lobe/emotional regulation and threat processing; ACC/reward and decision-making) (44). Moreover, children in lower SES groups also show a range of *functional* deficits, including in brain regions that support ECF, such as prefrontally-mediated attentional focus (49–51), and in prefrontal and parietal regions supporting working memory (52, 53). Lower SES is also associated with greater amygdala responsivity to threatening and fearful stimuli (e.g., faces) in adolescence (54). Interestingly, the functional networks between these cortical and subcortical regions appear to be disrupted by the experience of poverty, with low SES children showing reduced functional connectivity between cortical and subcortical regions during both task-oriented (i.e., emotional processing) and resting-state imaging paradigms (55–57). A recent analysis of the structural connectome in healthy children (6–11 years) found that lower income-to-needs ratios were predictive of greater network inefficiency, particularly for girls, in a range of SUD-relevant regions (e.g., cingulate, insula, amygdala), further supporting the notion that childhood poverty leads to widespread disruption of brain networks (58) and suggesting at least one potential environmental factor that may differentially contribute to risk between males and females. Collectively, these studies, while not explicitly considering substance use or misuse as an outcome, all point to a disruption of structural and functional neurodevelopmental trajectories for those who are economically disadvantaged in regions that are considered relevant for neurocognitive functions related to the extent of SUD liability. Importantly, the impacts of poverty are inextricably linked to the influences of stress and trauma on neurodevelopmental pathways that underlie the risk for substance abuse, since these experiences often occur in concert with one another. However, from a prevention perspective, it may be particularly advantageous to consider poverty as a key factor underlying a maladaptive risk pathway, since economic disadvantage can be more clearly—albeit not more simply—targeted via widely scaling appropriate, evidence-based interventions and policies.

As noted above, it is likely that a key factor underlying the impact of these types of environmental factors (i.e., CM, poverty) on SUD risk are epigenetic modifications that mediate gene-by-environment interactions, specifically those epigenetic factors involved in altering gene regulation of neurobiological systems that are relevant for maladaptive pathways that lead to SUD (59). Of note in the relationship between stress/trauma, neurodevelopment, and substance abuse liability is the role of micro RNAs (MiRNAs) (60). MiRNAs are short noncoding RNAs that epigenetically modulate gene expression. They also regulate central nervous system physiology and have the potential to contribute to alterations in complex systems, including dopaminergic and glutamatergic systems, which are both implicated in SUD (60). A particularly intriguing observation from preclinical studies of SUD-related behaviors is the phenomena of transgenerational epigenetic effects. For example, in rat models, adult drug taking that precedes conception appears to influence reward-related behavior and drug self-administration in first-generation offspring (61, 62). While these types of transgenerational impacts of SUD are potentially highly relevant for those families and communities that are at highest risk for SUD and for which effective prevention is most urgently needed, further study is required to demonstrate similar transgenerational mechanisms in humans. If such effects are found, this information may offer a particularly novel opportunity for cross-generational preventive interventions for SUD.

##### Neurological Development

The role of deviations or delays in neurodevelopmental pathways underlying problem (especially high risk) behaviors that often precede substance use has been increasingly recognized in studies of SUD risk. As in our Accumulative Risk Model, perturbations in brain structure and function are commonly viewed as critical mediators between the developmental context (i.e., relevant genetics and environmental factors) and the cognitive, behavioral, and affective phenotypes that precede problematic substance use. Understanding the neurobiological contribution to the etiology of substance use involves characterization of brain maturational processes that underlie neurocognitive development during critical periods of development, such as adolescence, that are associated with substance use (e.g., reduced inhibitory control and increased reward sensitivity).

While substance abuse is the result of maladaptive developmental trajectories with their roots in the prenatal period and lasting until the mid to late 20s, substance use initiation is most typical in early to mid-adolescence and, for the subgroup that escalates, substance abuse peaks during the transition into emerging adulthood (63). Critically, new social challenges facing adolescents (e.g., increased autonomous decision-making) coincide with complex changes in brain function and connectivity taking place throughout this time, which have implications for adaptive decision-making and the ability to self-regulate behavior and emotion (64, 65). In effect, some degree of impulsivity, risk-taking, and sensation seeking is normative during adolescence, as indicated above; however, a heightened level of risk-taking may extend from a combination of social circumstances and nonnormative neurodevelopmental immaturity or dysfunction.

Neurobiological development during adolescence occurs transitionally rather than as a single snapshot in time (66). The PFC, which is responsible for ECFs, such as decision-making, impulse control, and working memory, undergoes prolonged development and is still largely under construction during adolescence. A central role of ECFs is to promote behaviors that shield long-term goals from the temptations afforded by short-term benefits that often lead to negative consequences (67). Prefrontal “top-down” neurocognitive regulation over subcortical regions that support affective processes (e.g., emotion regulation, affective decision-making) is somewhat functionally disconnected throughout adolescence (68), translating into a natural bias in adolescents toward acting on emotional stimuli with relatively little cognitive control over those actions. Through both the natural course of development and environmental experience, connections between these regions are strengthened, providing a mechanism for increasing top-down regulation of emotional brain systems and improved behavioral outcomes (69, 70).

In addition, brain circuits involved in reward processing (e.g., the mesocorticolimbic pathway that involves typical reward-related regions, such as the ventral striatum) show rapid maturation during the adolescent years (71–73), which can have the effect of heightening sensitivity to rewarding experiences (i.e., making adolescents typically more reward sensitive and less risk averse). Paralleling this increase in reward sensitivity during this developmental period is a greater tendency toward sensation/novelty seeking (74). The developmental trajectory of reward circuitry likely plays a critical role in substance use initiation rates in early to mid-adolescence and may be especially pronounced in the subgroup that escalates use. Moreover, subsequent use of substances has the potential to exacerbate an already heightened reward sensitivity in some adolescents, resulting in a strengthening of the drug’s reinforcing properties (75).

Compounding these neurological liabilities (i.e., reduced ECF and heightened reward sensitivity) are early puberty and erratic hormone levels, as well as the potential to experience detrimental environmental conditions, such as stress, adversity, maltreatment, and other negative experiences that compromise neurodevelopment and can cause measurable dysfunction in these systems. Thus, regardless of the source of delayed or deficient neurodevelopment, the imbalance between increasing social demands and emergent neurobiological systems during adolescence may lead to heightened vulnerability to substance use and escalation (76). This evidence has direct implications for attempts to parse the developmental trajectories that give rise to SUD and the design of intervention components that effectively target this period of development.

##### Stress Exposures and Physiological Reactivity

“Stress” refers to processes involving perception, appraisal, and response to harmful, threatening, or challenging external events or conditions, known as “stressors,” such as poverty, prenatal exposures, child maltreatment, divorce, and bereavement (77). It is a major common denominator across the neurobiological and psychological domains discussed above and is a ubiquitous factor in susceptibility to substance use, escalation, relapse, and treatment resistance (78, 79). There is substantial evidence to support the role of stress in substance use trajectories [e.g., Refs. (6, 80)]; early life adversity is markedly associated with increased risk for substance use, abuse, and dependence (5, 81, 82).

Chronic and/or severe stress early in life alter emergent stress signaling pathways that, in effect, impair the ability of the PFC to exert cognitive control over more reflexive responses. For example, studies have shown neurodevelopmental deficits or delays in mesocorticolimbic circuits in adults who were maltreated as children, suggesting that functional aberrations may be due, in part, to dysregulation in this network of prefrontal and limbic regions (83, 84). Stress exposures also disrupt both hormonal and physiological systems that regulate these functions at the level of brain and peripheral nervous system, thereby impairing learning, memory, decision-making, and other functions that normally support self-regulation of behavior (85–87). Alterations in hormonal systems (e.g., cortisol) that modulate these functions (85) occur with chronically elevated levels of stress hormones which can reduce hippocampal volume, impair memory, and decision-making (2, 87). Psychophysiological studies also show effects of stress on autonomic responses such as heart rate that, when perturbed, are associated with psychopathology (88–90). In general, greater levels of stress alter brain circuitry, largely impacting the ability of the PFC to maintain behavioral and cognitive control over affective responses (91). These biological stress responses activate the same neural systems found altered in many mental health disorders and that underlie the rewarding effects of drugs (e.g., dopaminergic mesocorticolimbic circuitry), potentially reinforcing drug-taking behaviors (92, 93). As a result, when an individual experiences a great deal of stress or adversity, these stress responses affect brain function, leading to poor decision-making and other executive cognitive skills; thus, drug taking may occur as a maladaptive response to stressful experiences.

Adversity and stress have been inextricably linked to risk for substance abuse throughout adolescence (5, 6) possibly via effects on neurocognitive development in a way that predisposes individuals to impulsivity and externalizing behaviors (94, 95). In fact, numerous studies have demonstrated associations between increasing levels of emotional and physiological stress and decreases in behavioral control, heightened impulsivity, and greater incidence of maladaptive behaviors [e.g., Refs. (96–98)]. Moreover, a growing body of evidence suggests that impulsivity and externalizing behaviors may, in particular, mediate the association between adversity and risk for later substance abuse (99). These behaviors have also been consistently associated with deficits in ECFs (15, 100–102) and reportedly develop in response to exposure to early adversity [for review, see Ref. (43)]. As such, there is a plausible confluence of factors at play, corresponding to the Accumulative Risk Model, which may shed light on the delayed development of adverse outcomes; specifically, pathways from early adversity that interact with risk genotypes to impact emergent neural circuits and, in turn, externalizing and impulsive behaviors, thereby increasing propensity to substance misuse.

These findings suggest that very early development sets the stage for a heightened response to substances through primary biological, psychological, and social systems. Andersen and Teicher (103) provide evidence that early life stress predisposes individuals to abuse substances later via alterations in immature neurophysiological systems that have yet to come on board. In adolescence, when these emergent systems become increasingly functional, the damage is expressed in heightened risk for psychopathology. If the behavioral effects of early childhood stress are not observable until neural connections begin to onboard during adolescence (103, 104), implications for prevention are intriguing. For example, a few studies are now suggesting that training to reduce impulsivity, improve ECF, and integrate components that focus on “top-down” cognitive control has potential to reduce substance use initiation *and* escalation (105). Recognizing the increased risk for substance use in people who have experienced early life stressors is critical to guide prevention efforts designed to both prevent the exposure and counteract the potential subsequent negative consequences.

##### Cognitive and Behavioral Phenotypes

Externalizing disorders are consistently implicated in the use and abuse of a range of substances (106). The neurocognitive characteristics of children and adolescents with externalizing behaviors include heightened reward sensitivity, poor inhibitory control, aggression, and novelty seeking (107, 108). Variation in these dimensions, particularly impulsivity and reward seeking, contributes to the likelihood of substance use initiation as well as the transitions from initial to intermittent to regular substance use, the transition from abuse to addiction, and the propensity for repeated relapse after achieving abstinence (109). Individuals who measure highly on these traits tend to seek highly stimulating and risky situations and show less anxiety in anticipation of the consequences of their behavior (109, 110). Importantly, these cognitive and behavioral predispositions have differential impacts on substance use patterns at different developmental stages (111, 112). Normative development during adolescence is typified by heightened levels of impulsivity and novelty seeking, in part due to dramatic fluctuations in hormone levels that affect brain development and other systems modulating neurocognition (113). However, the subgroup of adolescents that exhibit heightened impulsivity and sensation seeking are at elevated risk to abuse substances (4, 114). These characteristics may, in effect, contribute to individual differences in the reinforcing effects of substances (115).

Psychopathology in many forms [e.g., posttraumatic stress disorder (PTSD), depression, anxiety, conduct disorder (CD), attention deficit hyperactivity disorder (ADHD), oppositional defiant disorder (ODD), antisocial personality disorder (ASPD)] is strongly and consistently related to the risk of substance abuse [for review, see Ref. (116)]. Individuals with these disorders are more likely to use/abuse substances and at an earlier age than those without such disorders (117, 118). They are also more likely to be resistant to substance abuse treatment (119). In general, individuals afflicted with mental health problems are often compromised in their ability to effectively meet social task challenges, as doing so requires intact neurocognitive functions, which are often compromised in psychiatric disorders (120, 121). Further compounding the risk, the development of mental health disorders increases use in an effort to manage symptoms and this association is likely to vary as a function of the type of mental health disorder. Mood and anxiety disorders, for example, double the risk for SUDs (122). Relatedly, alexithymia (i.e., an emotional processing deficit, whereby one experiences difficulty identifying or describing one’s emotions), has been identified as a pertinent risk factor for SUD (e.g., up to 2/3 of patients with SUD exhibit alexithymia) (123) and may increase the risk of negative outcomes such as suicide and self-harm in those who develop SUD (124–126). Since alexithymia predicts poor emotional regulation (127), which in turn predicts poor response to intervention [e.g., Ref. (128)], along with other forms of psychopathology that predict SU liability, it may be an important phenotypic characteristic to consider in the context of the differential trajectories highlighted here.

Gender is also an important factor in the association between SUD and other types of psychopathology. For example, males more often exhibit antisocial personality and conduct disorders (129), while females often have higher rates of mood and anxiety disorders (130); as a result of these gender-specific differences in prevalence of certain psychopathologies and their differential associations with substance misuse, these disorders confer differential gender-related risks for substance abuse (131).

#### Delayed Risk for Substance Misuse

The second maladaptive pathway toward substance misuse that is theorized here and conceptualized in our model is characterized as *delayed risk*: i.e., individuals who either initiate in adolescence but do not escalate until early/emerging adulthood, or who initiate and develop substance-related problems in late adolescence or early adulthood [e.g., Ref. (132)]. While understudied, delayed risk is also seen in those who do not develop SUD until middle or late adulthood [e.g., Ref. (133)], although the critical factors (especially environmental influences) that underlie such trajectories later in life may be distinct from those that drive misuse and escalation in earlier developmental periods.

Longitudinal studies have distinguished delayed risk in late adolescence/emerging adulthood by histories of externalizing behaviors, child maltreatment, and being bullied by peers, whereas other patterns of use (e.g., limited use) have been characterized by family instability and anxiety disorders (134). However, a nonlongitudinal study of men with substance abuse reported nearly identical childhood and adolescent risk factors (135), while another longitudinal study (136) found similar factors to be predictive of adolescent and adult illicit drug use, with the addition of early cannabis use as a significant catalyst for both groups, thus complicating our ability to disentangle which factors may be more closely coupled with delayed vs. early risk.

It may also be the case that the social transitions into emerging adulthood represents a significant risk factor for substance abuse in those who have difficulty with the novel demands of this developmental period. Though social role transitions (e.g., stable employment, marriage/cohabitation, parenthood) are typically associated with decreases in substance use (137), timing of, preparedness for, and adjustment to these transitions may be critical in predicting delayed risk for substance abuse. For example, in a longitudinal study of 18–33 year olds, an earlier transition into parenthood (i.e., late teens, early 20s) was associated with an increased rate of tobacco misuse (138). Likewise, high school seniors making the transition into early adulthood who have no plans for college are more likely to misuse prescription opioids compared to their peers who did have such plans (139). It is possible that the stress of newfound social demands and responsibilities for which some individuals are not developmentally prepared provides a generative context for substance abuse (138). In other words, there may be a developmental mismatch between expectations in adolescence for mature, autonomous behavior and their neurological, psychological, and social capacity for taking on a significantly greater level of responsibility during this transitional period. This mismatch may be an important predictor of delayed substance abuse, both during emerging adulthood and in later adulthood (140). Interestingly, the discordance between demands and abilities as adolescents approach adulthood may actually predict substance misuse later in adulthood. For instance, Green and colleagues (133) reported that individuals who were unmarried, unemployed, and had lower social integration during young adulthood were more likely to have delayed onset SUDs during middle adulthood. Taken together, these results suggest that individuals who experience difficulty adapting to developmentally normative social transitions, particularly during emerging adulthood, when there is less parental support, greater opportunities to engage in risky behaviors, and more access to substances, may be at increased risk for developing SUDs.

### Resilience and Resistance to Substance Misuse

Trajectories of *resilience* (i.e., rebounding from adversity after an initially altered trajectory or decline in functioning) and *resistance* (i.e., maintenance of adaptive functioning, despite adversity) are less well characterized than risk in the existing literature, for several reasons. First, since adversity and its negative consequences are major public health concerns, there has historically been a strong emphasis on deleterious sequelae of developmental adversity and stress, to the relative neglect of positive outcomes. Second, resilience and resistance are often not delineated as separate processes in the literature; that is, some proportion of those who are operationally defined as resilient may more aptly be defined as resistant. While studies typically characterize “resilience” as the absence of behavioral health or psychiatric disorders in adulthood, the majority do not track fluctuations in pathways over time and, thus, are unable to distinguish subgroups that sustain mental and behavioral health from childhood into adulthood relative to subgroups that respond to adversity with a decline in function but eventually improve; both classes will appear similar when outcomes are measured in adulthood. Thus, conceptualizing resilience as a single-end point (e.g., lack of psychopathology) may be misleading and prohibits the differentiation of subgroups that have followed pathways that may have diverged at various points in development. Finally, resistance is not often considered explicitly in the SUD literature. This is likely because characterizing subgroups *not* engaging in high-risk behaviors has been less of a priority and possibly also because this subgroup—which does not misuse substances or exhibit other forms of psychopathology—is not readily discernable, particularly in nonlongitudinal, cross-sectional studies. Consequently, the concept of resistance has largely not been in the SUD research lexicon, and has thus been almost entirely overlooked. A notable exception is a study by Hobfoll and colleagues (141) where they contrasted between resistance and resilience, both behaviorally and biologically, in individuals who experienced significant trauma and, yet, ultimately followed different pathways. These trajectories were identified and characterized in individuals who experienced ongoing terrorism. The authors suggested that resistance and resilience differ in terms of impact (resist vs. absorb), function (continue vs. gradually degrade), resumption of function (immediate vs. delayed), as well as overall response to adversity (defeat vs. limit).

While the conceptual model presented in this paper is focused on differentially characterizing trajectories on the basis of neurocognitive evidence, the paucity of literature clearly distinguishing resilience from resistance precludes such a review specifically for outcomes that are overall adaptive. Therefore, to explore the distinction between resilience and resistance further, we instead describe the few existing studies that delineate some of the relevant factors that likely contribute to and distinguish these two positive developmental pathways. Given associations between these influential factors, neurocognitive development, and functioning across the lifespan, we rely on these findings to formulate hypotheses regarding how these positive pathways may operate.

#### Neurocognitive Factors Relating to Adaptive Pathways

Despite overwhelming evidence of early stress and trauma’s adverse influences on adult outcomes, many individuals exposed to trauma exhibit healthy adult functioning [e.g., Refs. (142–144)]. Some studies have begun to highlight the potential of strengthening cognitive and emotion regulatory skills to act in a protective capacity in those who have experienced trauma. For example, in a study of highly traumatized urban adults compared those who did or did not exhibit psychopathology, those who did not develop psychopathology had better nonverbal memory than those who did, despite similar levels of CM and trauma (145). Other work has suggested that emotion regulation, which is related to impulsivity and subsequent substance use patterns (146), is predictive of extent of adaptive coping in maltreated children (147). Moreover, in children exposed to political violence, higher levels of cognitive flexibility has been shown to moderate the relationship between violence exposure and psychological well-being (148). Though these studies did not explicitly measure substance use, it is possible that having well-developed neurocognitive skills (e.g., memory, cognitive flexibility, emotion regulation) enables individuals who have experienced trauma to adaptively navigate their environments and avoid substance misuse and eventual dependence, despite a history of adverse experiences. Indeed, deficits in these neurocognitive skills are related to substance abuse (149–151), providing further support for the inverse relationship, with more robust neurocognitive skills predicting a decreased likelihood of developing SUDs.

Interestingly, a few studies suggest that early adversity may drive neurocognitive adaptation in some individuals in ways that enables them to outperform healthy controls or those who have had fewer adverse experiences. For example, Nolin and Ethier (152) reported that children who had a history of neglect evinced better planning and problem-solving skills than children without histories of abuse. There are also similar findings from research with older adults (i.e., 50 years and older) who have experienced CM, providing additional evidence of preserved cognitive functioning in spite of adverse experiences (e.g., visual memory, verbal memory, executive functioning, attention, processing speed) (153, 154). For example, Feeney and colleagues reported that older adults who had experienced childhood sexual abuse had better executive functioning, attention, and processing speed than those without maltreatment history (153). Similarly, another study demonstrated that, compared to those with moderate levels of CM, individuals with severe levels of maltreatment had lower risk of cognitive impairment (i.e., visual memory, executive functioning, and verbal memory) later in life (154).

Taken together, this work supports the concept of a subset of individuals who may have protective assets, particularly in neurocognitive domains of functioning, that enable them to thrive despite experiences of adversity, trauma, and stress. The extent to which their adaptation corresponds to resilience vs. resistance pathways in patterns of substance use remains to be explored. However, we posit that, based on indices of neurocognitive functioning, classes of individuals may be more aptly characterized by longitudinal investigations that aid in the delineation of critical time points corresponding to these divergent developmental pathways. In particular, a clearer understanding of adaptations to adversity will emerge with further investigation into resilient and resistant trajectories that correspond to the behavioral and mental health endpoints of interest. Longitudinal observations will allow us to more fully characterize adaptations, which are important predictors of ultimate outcomes (adaptive vs. maladaptive) and that may fluctuate or be sustained at particular developmental time points. As such, future work characterizing these different developmental pathways is critical for understanding the precursors of these trajectories and how they unfold *and* to identify and bolster neurocognitive factors that confer resilience or resistance.

#### Neuroimaging Correlates of Adaptive Pathways

A few recent neuroimaging studies have begun to pinpoint brain regions that differentiate trauma exposed individuals who do or do not exhibit adaptive outcomes (e.g., based on psychopathology or adaptive functioning status). For example, compared to those who experienced maladaptive outcomes, trauma-exposed youths who exhibit adaptive functioning have been found to have lower resting-state functional connectivity (rsFC) within default mode, salience, and executive control networks (155). Interestingly, all of these networks have been shown to be disrupted in substance-abusing samples (156, 157). Other rsFC studies have highlighted the dorsal ACC (dACC) as a region showing distinguishable patterns of connectivity in adaptive vs. maladaptive outcomes for those who have experienced early life stress and/or trauma (158, 159). For example, Philip and colleagues found increased rsFC between the thalamus and dACC in adults who experienced early adversity *without* psychiatric disorders compared to those with psychiatric disorders (158). These findings are intriguing with respect to potential neural correlates of resilient and resistant pathways pertaining to substance use, given that previous work has reported diminished activation and connectivity patterns in the dACC in substance-dependent individuals, particularly when processing rewards (160, 161).

Findings from several other neuroimaging studies suggest that the structure and function of the frontal lobe (e.g., volume, activation, connectivity) is implicated in adaptive functioning following adversity (162–164). Specifically, one study found that adaptive adolescents who had experienced early adversity had increased middle frontal and superior frontal gyri volumes compared to maladaptive adolescents who had experienced early adversity as well as those who had not experienced adversity (163). Moreover, the same study reported that middle frontal gyrus volume negatively correlated with problematic drinking in adolescents who were deemed adaptive but experienced early adversity (163). Another study found that compared to individuals with PTSD, those who were also trauma exposed but did not have any psychiatric disorders showed enhanced ability to recruit frontal regions associated with top-down attentional control during an emotional Stroop Task (165). Similarly, patterns of increased frontolimbic connectivity seem to distinguish maltreated individuals from healthy controls who were comparable in adaptive functioning, including a lack of substance abuse (164). Although these studies did not all measure substance use or neurocognitive functioning, they do provide initial support for increased volume and functional recruitment of frontal regions as being a neuroprotective factor in individuals who have experienced early adversity. Such findings are promising in their ability to distinguish neural profiles of adaptive and maladaptive traumatized populations; however, they also evoke many questions about how frontal lobe development progresses in individuals who follow resilient or resistant pathways in response to adversity. For instance, future studies could probe how specific neuroprotective factors (e.g., increased or decreased frontal lobe activation and connectivity) interact with other factors (e.g., genetic or environmental liabilities) to confer a likelihood of following a resilient or resistant pathway subsequent to early adversity.

#### Delineating Resilience and Resistance: Future Work

By and large, the literature points to several neurocognitive factors that likely contribute to resilience or resistance pathways subsequent to adversity. However, as noted, prior research has not made concerted attempts to disentangle these pathways, their precursors, and their trajectories. Therefore, many open questions remain as to how subgroups who attain successful outcomes following trauma, maltreatment, or other environmental adversities rebound from or, in contrast, resist engaging in substance misuse. Since not all survivors of adversity develop SUDs or other forms of psychopathology, it is critical for future work to pinpoint and characterize these subgroups. Moreover, the preliminary evidence cited above suggests that individual differences in neurocognitive skills or patterns of connectivity in regions of interest for SUDs may differ across development but may still ultimately predict similar adaptive outcomes. For instance, it is plausible that individuals who are less adept at regulating emotions and engaging executive functions (i.e., regulating top-down processes) may experience initial developmental disruptions that lead to substance use that they rebound from (i.e., resilience trajectory). In contrast, those who are more adept at these neurocognitive skills may resist substance use altogether (i.e., resistance trajectory). As others have suggested in the literature, resilience to adversity is a dynamic, state-like process, not simply a trait, and individuals who appear adaptive later in life may or may not have experienced initial maladaptive pathways from which they have rebounded. Recent studies have also proposed novel models [e.g., the *Resilience Portfolio Model* (165) or the *Diversity Portfolio Model* (166)] that conceptualize “resilience” as an arsenal of protective factors associated with healthier outcomes following trauma. Accordingly, the density and/or diversity of available protective resources and assets may shape their long-term capacity to adapt and thrive despite adverse experiences. As such, future studies that thoroughly characterize neurocognitive profiles, across the developmental timeline, and which delineate how such profiles interact with other factors known to bolster adaptive functioning, may be able to meaningfully distinguish those who are resilient and rebound from those who are resistant. This distinction in pathways is crucial, as those who are resilient may be categorized by particular vulnerabilities during specific windows of time that may serve as critical opportunities to successfully intervene with prevention programs. In summary, delineating the neurocognitive profiles of individuals who exhibit resistant vs. resilient pathways may be critical for identifying novel ways to bolster functioning in those who experience maladaptive pathways/outcomes.

## Methodological Approaches to Distinguish Trajectories

While there is convincing evidence for distinctions between risk, resistance, and resilience trajectories based on phenotypic presentations, studies have yet to effectively delineate the possible neurocognitive correlates or underpinnings that support their distinctions. This information may have important implications for more precision-based, developmentally sensitive intervention targeting. It is reasonable to surmise that environmental risk and protective factors may impact neurocognitive development in unique ways across individuals and/or subgroups, leading to different phenotypic outcomes. With respect to positive outcomes such as resilience, resistance, and recovery, this assumption is supported by the equifinality of the result—i.e., a similarly adaptive outcome profile across these different trajectories—and thus may be logistically difficult to differentiate. Cross-sectional research designs are inadequate in this endeavor; they may be able to confirm that various outcomes are predicted by the level of neurocognitive functioning at a single time point but they are unable to chart the dynamic interplay of risk and protective factors that impact the course of neurodevelopment and its relationship to final outcomes. In contrast, by establishing temporal ordering within subjects, longitudinal research designs are uniquely positioned to pinpoint developmental phenomena and their divergent pathways. Thus, a longitudinal approach is able to model the experiential and contextual impact on neurobiological factors across development to understand the nature of the various pathways that lead to eventual maladaptive versus adaptive outcomes. Pinpointing neural markers that distinguish individuals who move along these distinct pathways will help us to identify novel targets for intervention. By fully characterizing and differentiating these trajectories, longitudinal studies have the potential to aid in the delineation of the precise nature of influential factors at optimal time points along their development (e.g., adversity onset, treatment onset, redirection) and, in doing so, to identify malleable targets that exist along these trajectories, which will serve to maximize the translational potential of this research.

Latent class modeling has the potential to substantially aid in the determination and delineation of unique pathways that underlie SUD liability, including risk, resilience, and resistance. Latent class modeling refers to a group of statistical methods aimed at identifying unobservable (*latent*) subgroups within a particular population. It includes latent class analysis (LCA), which considers outcomes at a particular time point (e.g., adolescence), and a related methodology, latent transition analysis (LTA), which facilitates estimation of transition between subgroups over time. An application of LCA that includes consideration of the types of risk- and resilience/resistance-relevant factors outlined in the Accumulative Risk Model (**Figure 1**) and, especially, pertinent neurodevelopmental factors (e.g., neurocognitive processes, variation in brain structure, function, and connectivity) will facilitate the determination of which specific constellations of factors give rise to which intermediate phenotypes and associated pathways. Moreover, an LTA approach will allow us to determine which factors are particularly relevant at the time points where we see real or apparent shifts in developmental trajectories, either toward or away from increased liability and adverse outcomes.

These latent class approaches hold considerable potential for determining opportunities and methods to optimize preventive interventions. However, to-date, there is a relative paucity of research using latent class modeling in the context of risk for substance abuse and dependence that has focused on neuro-related factors and/or on the types of longitudinal approaches to SUD liability that we are suggesting here. Nonetheless, application of latent class models to substance abuse risk and treatment have revealed some interesting outcomes regarding how patterns of use may impact substance use behaviors or brain activity [e.g., Refs. (167–169)] and support the appropriateness of these methods in the context of SUD liability pathways.

## The Potential for Prevention

Based on a burgeoning body of evidence, brain development and function are, for better or for worse, clearly experience dependent. For worse, adversity in its many forms has the potential to impact neurodevelopmental trajectories in ways that undermine emergent self-regulatory mechanisms, increasing risk for psychopathology, including eventual SUD. However, for the better, the brain’s substantial plasticity translates to the potential for well-conceived prevention strategies to improve behavioral and mental health outcomes by positively impacting the same neurodevelopmental pathways. Although most prevention science studies do not attempt to elucidate the neural mediators of intervention responses, a considerable number of prevention programs have been shown to reliably reduce risk for substance abuse. Research to enhance our understanding of the neurodevelopmental effects of prevention programming has potential to further differentiate the pathways involved in the relationship between risk factors and behavioral outcomes and, in doing so, will identify mediating mechanisms that explain outcome heterogeneity. This argument is particularly compelling given that, at present, the evidence-based programs that have emerged from various disciplinary perspectives produce only small to modest effects on the phenotypes predictive of SUD risk and resilience/resistance pathways, as well as SUD itself. More comprehensive and in depth information is needed to advance predictive analytics and increase the precision with which we target programmatic components.

It is likely that evidence-based programs work at the level of the brain, driving adaptive changes in brain structure, function, and connectivity. Programs that focus on socioemotional and cognitive functioning are strong candidates in this regard. Development of these skills, both behaviorally and neurobiologically, are particularly vulnerable to adverse psychosocial and environmental influences. Programs that redirect and possibly normalize these specific dimensions of a child’s developmental pathway may exert a potent impact on corresponding behavioral, emotional, mental, and physical (e.g., brain function and fitness) domains. The effects of appropriately targeted interventions may be particularly remarkable for children who are disadvantaged by poverty and other social ills. Research that integrates multiple disciplines to better understand influences and outcomes related to substance abuse have directed us toward solutions for these problems that target underlying mechanisms and not solely the distal outcome of substance abuse, *per se*. In other words, it is vital that we address the factors that eventually lead to drug abuse prior to its development, the key principle behind prevention science.

The integrity of the way in which the brain develops in children is a prerequisite for adaptive responses to socioenvironmental challenges and thus, to favorable responses to intervention [e.g., Ref. (170)]. Thanks to the vast brain plasticity throughout childhood and adolescence, there is a great deal of variability in the way children develop in response to environmental inputs, including the divergent pathways under discussion here. This plasticity throughout early childhood and adolescence offers several optimal windows of opportunity for intervention. When neurodevelopment is on course or shows a trend toward improvement, overall intervention outcomes are likely to be favorable. In contrast, existing or emergent neurodevelopmental deficits or delays may compromise intervention effects, potentially explaining differential outcomes in response to even the most highly regarded and efficacious programs. A comprehensive evidence-based set of solutions (programs and policies) to prevent psychopathology and eventual drug abuse that operates to enhance developmental indicators of brain function in multiple domains are needed. This approach will, in turn, improve the ability to self-regulate behavior and reduce the risk for developing SUDs.

Applying this integrative and developmental perspective will lead to significant advancements in our ability to prevent substance use and the eventuality of SUD for some. Indeed, SUD intervention researchers have begun to incorporate cognitive training, mindfulness approaches, behavioral and environmental modifications, and other innovative strategies that target malleable neurodevelopmental processes that contribute to substance abuse (171, 172). Determining which early influences are particularly relevant will be critical to designing interventions that target the underlying generators of SUDs, before behavioral problems and substance use patterns become entrenched. And while there are many outstanding questions in this line of research, we do know enough about prevailing conditions that influence risk for SUDs to exert a positive impact now.

## Conclusions

Studies on the successes and failures in the treatment of SUDs are benefitting from the inclusion of neuroimaging, leading to the identification of biomarkers of SUDs and increasing our understanding of variability in treatment outcomes. Proximal biomarkers in prevention studies are similarly needed to provide targets for intervention, detect differentially receptive subgroups, predict intervention response, and broadly improve outcomes. This technique could be particularly promising for “proven” prevention strategies with protective longitudinal results from early childhood through adolescence and adulthood, but were created before the explosion of biomarker research. Important advances in studies including neuroimaging and other biomarkers have revealed activity within relevant neural circuits in association with behavioral change reflective of protection from substance abuse. The application of early neuroimaging to well-established prevention strategies has potential to elucidate the neural correlates of dimensions of functioning commonly implicated in substance use and related disorders, such as impulsivity, reward sensitivity, and cognitive control, among others. While these dimensions of functioning have been related to substance misuse, SUD treatment outcomes, and relapse, a better understanding of these dimensions and their neural correlates and how they correspond to the distinct adaptive and maladaptive developmental trajectories considered here (i.e., *risk, delayed risk, resilience*, and *resistance*) could identify malleable brain–behavior biomarkers for improving preventive intervention effects. Extending models from treatment research to prevention is sorely needed by identifying functional, malleable mediators, and moderators of well-established prevention programs. Indeed, this line of research—to identify biomarkers and conditions within which they interact that distinguish between developmental pathways—has potential to identify novel targets for intervention. Such information will provide curriculum developers with data critical to optimizing programs and compelling public, mental health, and educational policies to further scale effective prevention strategies. In effect, improving our ability to disrupt pathways to SUD would constitute a significant public health advancement with potential for population level effects.

## Author Contributions

DF initiated the concept for the paper. DF and ER conceived of the framework and ER elaborated on a proposed new model for understanding distinctive pathways to substance misuse and addiction, including pathways that eventually diverge and lead to positive outcomes despite prevailing risks. By and large, DF and ER framed and wrote the majority of the paper. ER and GP constructed the figures. GP contributed to the writing, referencing, and reviewing/editing of the paper.

## Funding

This work was supported by the National Institutes of Health, Eunice Kennedy Shriver National Institute of Child Health and Human Development, P50HD089922 (to GP and ER).

## Conflict of Interest Statement

The authors declare that the research was conducted in the absence of any commercial or financial relationships that could be construed as a potential conflict of interest.
