# Corrigendum: Neurocognitive Precursors of Substance Misuse Corresponding to Risk, Resistance, and Resilience Pathways: Implications for Prevention Science

**DOI:** 10.3389/fpsyt.2020.00057

**Published:** 2020-02-14

**Authors:** Emma J. Rose, Giorgia Picci, Diana H. Fishbein

**Affiliations:** ^1^ Program for Translational Research on Adversity and Neurodevelopment (P-TRAN), The Edna Bennett Pierce Prevention Research Center, The Pennsylvania State University, University Park, PA, United States; ^2^ Department of Human Development and Family Studies, The Pennsylvania State University, University Park, PA, United States

**Keywords:** neurocognitive, neuroimaging, substance misuse/abuse, risk, resilience, resistance, prevention science

In the original article, we neglected to include the funder National Institutes of Health, Eunice Kennedy Shriver National Institute of Child Health and Human Development, P50HD089922, to Giorgia Picci and Emma Rose.

The authors apologize for this error and state that this does not change the scientific conclusions of the article in any way.

